# Effect of soybean expeller supplementation during the final phase of sow gestation on litter birth weight

**DOI:** 10.14202/vetworld.2020.1245-1250

**Published:** 2020-07-02

**Authors:** Santiago Masi Mignaco, Ana Alba-Casals, Alicia Carranza, Julián Parada

**Affiliations:** 1 Departamento de Patología Animal, Facultad de Agronomía y Veterinaria, Universidad Nacional de Río Cuarto, Río Cuarto, Argentina; 2 IRTA, Centre de Recerca en Sanitat Animal (CReSA, IRTA-UAB), Campus de la Universitat Autònoma de Barcelona, Barcelona, Spain; 3 The OIE Collaborating Centre for the Research and Control of Emerging and Re-emerging Diseases in Europe (IRTA-CReSA), Barcelona, Spain; 4 Consejo Nacional de Investigaciones Científicas y Técnicas (CONICET), Córdoba, Argentina

**Keywords:** organic farms, piglet weight, production performance, sow nutrition

## Abstract

**Aim:**

Nutrition plays a key role in the production of pigs, especially in pregnant sows, where modifications in nutritional requirements can affect their productive performance. The aim of this study was to evaluate nutritional supplementation with soybean expeller in sows during the last third of the gestation period and its effect on litter birth weight.

**Materials and Methods:**

A quasi-experimental study was conducted on a farrow-to-finish farm, where 192 sows were equally assigned to treatment and control groups. Several variables were recorded at both the sow and piglet level. The treatment group consisted of piglets from 95 sows supplemented with soybean expeller during the final phase of gestation (20 days), and the comparison group consisted of piglets from 97 sows fed only with a commercial balanced ration (control group).

**Results:**

Soybean expeller supplementation increased individual piglet weight by 190-270 g, and the increased number of live piglets could decrease the weight of each piglet. Moreover, the number of piglets weighing <900 g decreased by 10% as compared to the control group, indicating that supplementation could improve pre-weaning mortality.

**Conclusion:**

Our results suggest that soybean expeller supplementation in sows during the last third of the gestation period could improve production performance, especially on organic farms.

## Introduction

Nutrition plays a key role in the production of all animal species. The nutritional management of pregnant sows on traditional and organic farms is usually limited to supplying the same feed throughout the whole period of gestation and adjusting the amount provided based on the week of pregnancy. However, this method does not take into account that the exponential growth of piglets during the last third of gestation modifies the nutritional requirements of sows [1]. An important relationship between nutrition at this stage and litter performance at farrowing has been proposed by several researchers [2,3], even accounting for variation due to the number of parities (NP) of the sow [4]. However, in some studies [5], variations in feed intake during gestation were more beneficial for sows than for piglets. With regard to the last third of gestation, a previous study demonstrates the relationship between increased feed supply during this period and improved embryo viability and even peripartum variables [6]. However, this theory remains controversial. Other studies debate its usefulness and have even demonstrated an opposite effect [7,8]. These differences in results can be explained by the variability of factors such as sow parity, gestation time, and food nutrients and their effect on muscular and nervous tissue growth in piglets [9]. The importance of piglet weight at birth and its effect on mortality and performance during fattening has been broadly demonstrated by several authors [10,11].

A wide variety of nutrients and food components have been proposed as limiting factors for piglet development and female productivity during the final phase of gestation [1]. Among these, levels of essential amino acids [12,13] and energy [14] have been most frequently studied. However, micronutrient-specific supplementation is often difficult to apply and is not permitted on organic farms; thus, an alternative feed strategy, such as the use of soybean expeller, could improve productive performance in these situations.

The aim of this study was to evaluate nutritional supplementation with soybean expeller in sows during the last third of the gestation period and its effect on litter birth weight.

## Materials and Methods

### Ethical approval

The study was carried out in accordance with the guidelines laid down by the Research Ethics Committee of the National University of Río Cuarto.

### Study design, location and period

To evaluate the effect of the nutritional supplement on the performance of the piglets, a ­quasi- experimental study was conducted on a commercial farm located in Córdoba with 220 sows between May and September of 2016. This site is a fully confined farrow-to-finish farm with a known history of good production performance and health status.

### Data collection

We recorded data on litter performance from two different groups of sows over 20 consecutive weeks. The treatment group consisted of piglets from 95 sows supplemented with soybean expeller during the final phase of gestation (20 days), and the comparison group consisted of piglets coming from 97 sows fed only with a commercial balanced ration (control group). To control for confounding extraneous factors, each weekly breeding group was assigned to the control or treatment group.

All females belonged to the same genetic line and were considered for this study at the 90^th^ day of gestation. The sows were housed in a barn with natural ventilation in individual stalls (2 m × 0.6 m). The feeding system was fully automated with individual stall regulators. All sows were fed twice a day with a gestation-balanced ration. Beginning on the 90^th^ day of gestation, the amount of feed offered in both groups was increased to 2.8 kg/day, and in the treatment group, 400 g of extruded and pressed soybean expeller (of known nutritional composition) was added once a day with the morning feeding.

The feed was produced on the farm and was stored in a silo outside the gestation barn. Maize was produced on the farm and was analyzed for nutritional control, as well as for mycotoxin levels, which were acceptably low.

During the study period, the soybean expeller came from the same supplier. Its nutritional composition was analyzed by wet chemistry and ­near-infrared spectroscopy, which allowed us to determine the nutritional composition of the base diet and the soybean expeller supplemented diet (Table-1).

**Table-1:** Daily nutritional intake for each sow from the 90^th^ day of gestation either from the control group (base diet) or the soybean expeller supplemented group (treatment).

Feed Composition	Base diet	Treatment
Protein (g)	404	546
Metabolizable energy (kcal)	8859	10,196
Digestible lysine (g)	16.8	25.6
Digestible arginine	22.4	33.8
Calcium	24.1	24.9
Phosphorus available	11.2	12.0

At farrowing, live-born and stillborn piglets were counted to determine the total number of animals per litter. In the calculation of the number of stillborn piglets, the number of live-born animals that died shortly after birth was not considered. Each piglet was weighed using a calibrated electronic scale with a maximum of 15 kg and an error of 0.005 kg after being attended by a midwife. Adequate measures were taken to minimize pain or discomfort during the procedure.

Diverse variables were recorded at the sow and piglet level. At the sow level, the following variables were collected: Individual identification of the sow, type of nutritional treatment (NT) administered, NP, litter weight on average, and number of live-born and stillborn piglets within the litter. At the piglet level, the variables collected were individual identification of the piglet, type of NT administered to the mother, individual weight of the piglet at birth, number of piglets born alive (BA) and stillborn within the same litter, and NP in which the piglet was born.

### Statistical analysis

The process of analysis was comprised of different steps. Initially, to detect and correct possible errors in the raw data, the data were pre-processed. Then, an exploratory descriptive analysis was carried out to summarize the main traits of each variable, assess their distribution, formulate hypotheses, identify potential confounders, and design appropriate models to assess the influence of the soybean expeller supplement administered to the sows on the piglet performance at the litter and piglet level. With this goal, the normality of each variable was assessed visually and tested using a Shapiro-Wilk test. Finally, to quantify the influence of the NT in sows on the performance of the piglets, parametric and non-parametric bivariate analyses were conducted according to the distribution of each variable, and two explicative models were proposed. First, the influence of feeding this supplementary diet was analyzed by average weight at the litter level. Here, a multiple linear regression was used, taking into account the initial explanatory variables “the group of nutritional treatment,” “the number of parities,” and “the number of piglets born alive,” along with their possible interactions. The variables included in the final model were selected based on a manual stepwise procedure using the lowest Akaike Information Criteria (AIC). To validate the model, a diagnostic checking of its residuals was conducted to guarantee heteroscedasticity and normality and to identify possible influential observations.

Next, the influence of the NT was assessed based on the weight of each individual piglet using a mixed linear model. This model included NT, NP, and BA as fixed factors, with possible interactions between these factors and “the mother sow” as a random effect. The variables included in the final model were also selected based on a manual stepwise procedure using the lowest AIC.

All statistical analyses and visual methods were performed using the base R program (R Development Core Team, 2015) and the lattice [15], ggplot2 [16], and lme4 [17] complementary packages.

## Results

**Figure-1:**
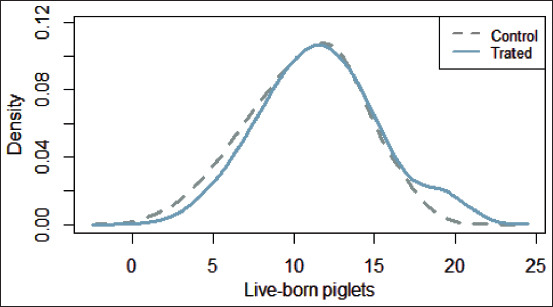
Distribution of live-born piglets according to the group of nutritional treatment.

During the study, 192 farrowing sows were analyzed: 97 in the control group and 95 in the treatment group. A total of 2142 live-born piglets were analyzed during the study: 1039 in the control group and 1103 in the treatment group. The number of piglets BA followed a normal distribution in both groups (Figure-1), with a mean of 10.7 piglets (SD 3.3) in the control group and 11.6 (SD 3.5) in the treatment group. However, the difference between the two groups was not statistically significant (Welch two-sample t-test with t=−1.79 and p=0.075).

Seventy-two stillborn piglets were recovered from the 97 farrowing sows in the control group, representing an average of 5.75% of the litter, and a median of 0 (interquartile range [IQR] 1) piglet. In the treatment group, 94 stillborn piglets were recorded from the 95 farrowing sows, for an average of 7.35% of the litter and a median of 1 (IQR 1) stillborn piglet. The distribution of this variable was not normal, and the differences between the medians were slightly significant using a Mann–Whitney U-test, non-parametric test (p=0.042).

**Figure-2:**
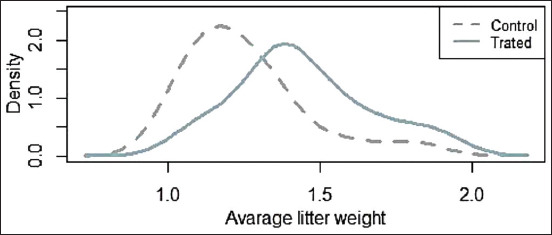
Density plot of the average litter weight from treated and untreated (control) sows.

The “individual piglet weight” did not follow a normal distribution. In the control group, the median was 1.2 kg, with a minimum of 0.75 kg and a maximum of 2 kg. In the piglets from the treatment group, the median was 1.4 kg, the minimum 0.8 kg, and the maximum 2.5 kg. Using a Mann–Whitney U-test, statistically significant differences were observed between piglets from sows fed with or without soybean expeller supplementation (p<0.01). Similar results were obtained by comparing the average litter weight (Figure-2).

The final model proposed included the NT and the interaction between BA and NP as explanatory variables (Table-2) and could be expressed as follows:

Average weight litter_j_ = Intercept + βtNT_j_ + β_tp_BA: NP_j_ + ϵ_j_,

with ϵ_j_ being the model error for the subject j, where ϵ_j_ ~ N(0,σ2).

**Table-2:** Results of the linear model for average weight litter and weight piglet, in relation with NT, NP, number of BA piglets, and the interaction between BA and NP.

Average weight litter_j_ = Intercept + βtNT_j_ + β_tp_BA : NP_j_ + ϵ_j_				

Variable	β	S.E. β	95% CI	p
Intercept	1.501	0.093		
NT	0.227	0.023	0.182/0.273	<0.001
NP	0.041	0.020	−0.0003/0.082	0.051
BA	−0.027	0.008	−0.044/−0.011	0.001
NP:BA	−0.002	0.001	−0.006/−0.001	0.145
Weight piglet_ij_ = Intercept + βtNT_j_ + β_p_NP_s*_BA_j_+ 1 | Sow_i_ +ϵ_ij_				

**Variables**	**Variance**			**Std. Dev**

Random effects				
Sow	0.021			0.145

**Variable**	**Variance**	**Std. deviation**		**95% CI**

Fixed effects				
Intercept	1.712	0.095		1.513/1.911
NT. control	−0.228	0.022		−0.267/−0.186
NP	0.044	0.021		−0.0004/0.008
BA	−0.026	0.008		−0.044/-0.008
NP:BA	−0.003	0.001		−0.006/0.0008

NT=Nutritional treatment, NP=Number of parity, BA=Born alive, CI=Confidence interval

**Figure-3:**
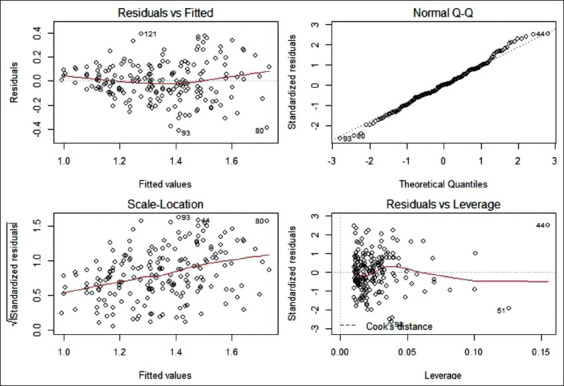
Diagnostic plots of the residuals checks of l-model for weight of live-born piglets.

**Figure-4:**
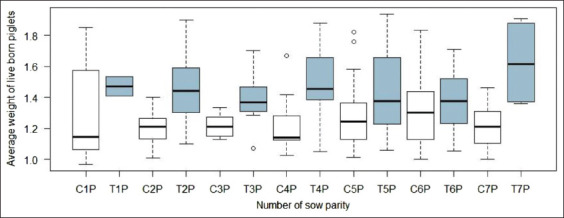
Average weight of live-born piglets according to number of parity of the sow in treated (T) and control (C) groups.

The confidence intervals of this model indicated that the soybean expeller supplementation in sows was associated with an increase in average weight between 180 and 270 g. In addition, the number of piglets BA had a slight influence on the average weight of the litter. Figure-3 shows the diagnostic plots of the residual checks. No statistical differences were observed in the weight of live-born piglets among the different parity numbers of sows from the studied groups (Figure-4).

To assess the effect of the soybean expeller supplementation in sows on the weight of each individual piglet (Table-2), the final mixed linear model was as follows:

Weight piglet_ij_ = Intercept + βtNT_j_ + β_p_NP_*_BA_j_ + 1 | Sow_i_ +ϵ_ij_

Here, the results showed that the soybean expeller supplementation increased the individual piglet weight by 0.19-0.27 kg and that the higher number of live piglets could decrease the weight of each piglet. In addition, the results show that weight of each piglet was significantly influenced by the effect of each sow.

## Discussion

Soybean expeller supplementation in sows during the last third of gestation increased the weight of live piglets from treated sows by more than 200 g as compared to the control group. According to a previous study [1], this difference could be due to the increase in the supply of essential amino acids such as arginine in the diet. Other possible explanations in the literature point to lysine as being responsible for this improvement in weight since it is a strictly essential amino acid not synthesized by pigs [12] and is of great importance for the development of both primary and secondary muscle fibers [7,18]. This was demonstrated by a previous study [19], which reported that under a diet with high inclusion of lysine, sows presented higher piglet at weaning and better nutritional composition of colostrum and milk, especially with regard to the amount of fat and protein.

Although this study did not use synthetic arginine or lysine but opted for cheaper and easier to apply supplementation, which makes it difficult to compare with the results obtained by the cited authors, knowing the nutritional composition of the soybean expeller used can provide a framework for comparison. The 400 g of supplement contributed 0.40% more arginine and 0.31% more lysine to the daily intake as compared to the control group diet, which could explain the increases in the performance of piglets from treated sows in light of the previous studies.

An increase in piglet weight has also been associated with the use of high-energy diets [14], where the increase was slightly over 1000 Kcal/day in the supplemented sows. However, in other studies [20], the energy increase in the diet did not necessarily affect the weight of piglets, but did affect the body condition of the sow. According to one previous study [5], an increase in the amount of gestation balanced ration in the last third of gestation was more beneficial for the sow than for the piglets since differences were not observed in the weight of the litter in sows fed more than 3 kg compared to sows fed <3 kg. This suggests that an increase in the amount of ration is not sufficient *per se* to impact the piglets, but that a change in the composition of the formula is necessary.

It is important to note that piglets with a birth weight of less than 900 g have 60% lower rate of survival compared to heavier piglets [2]. In the present study, the percentage of piglets below that weight was lower in the treatment group (4%) than in the control group (13%). Therefore, although they were not measured in the present study, it could be inferred that under these conditions, pre-weaning mortality and possibly the number of weaned piglets per sow per year would improve. This is supported by other studies [21] showing that weight is a determining factor in the survival of piglets at farrowing because it influences the vitality of the piglets as well as the thermoregulation capacity and growth of the newborns. In addition, low birth weight reduces the mobility of the piglet due to lack of maturation of the nervous tissue, thus compromising suckling and increasing the odds of crushing [9]. Moreover, it might improve the growth performance of the herd because the higher the birth weight, the greater the daily weight gain of these animals at maternity as well as post-weaning and during fattening [10].

On the other hand, in the present study, the magnitude of the weight increases of the piglets at birth decreased with each unit of increase in the number of piglets per litter, a result that was supported by the previous studies [10,11,22]. In addition, although the treatment increased the average weight of the piglets, the within-litter birth weight variation was also greater. This greater dispersion of weight has been associated with an increase in perinatal mortality due to unequal competition for resources between lightweight and heavier piglets, which occurs even in prenatal life. This could explain the differences observed in the number of live-born and stillborn piglets in the treated sows compared to the controls in the present study, which differed from the previous studies showing that supplementation during the end of gestation decreased the number of stillborn piglets [13]. Finally, in the present study, no significant differences were observed in the number and weight of live piglets based on the number of sow parities.

## Conclusion

According to our results, the administration of a nutritional supplement with soybean expeller to sows during the last third of gestation improved the weight of piglets at birth, and the magnitude of the increase was related to the treatment and the number of live piglets in the litter. Our results suggest that soybean expeller supplementation in sows might help to improve production performance, especially on organic farms.

## Authors’ Contributions

SMM, AC, and JP designed the experiment. SM collected data and performed the experiment. AA and JP made the statistical analysis. All authors were involved in the writing, analysis of the data, and reviewed the manuscript, and they approved the final manuscript.

## References

[1] De Vos M, Che L, Huygelen V, Willemen S, Michiels J, Van Cruchten S, Van Ginneken C (2014). Nutritional interventions to prevent and rear low-birthweight piglets. J. Anim. Physiol. Anim. Nutr.

[2] Opschoor C, Bloemhof S, Knol E, Willems E (2012). The Economic Benefit of Heavier Piglets:Relations Between Birthweight and Piglet Survival and Finisher Performance. International Pig Veterinary Society Congress, Korea.

[3] Muns R, Nuntapaitoon M, Tummaruk P (2016). Non-infectious causes of pre-weaning mortality in piglets. Livest. Sci.

[4] Gómez-Carballa F, Lara L, Nieto R, Aguilera J.F (2013). Response of the Iberian sow to protein supply and feeding level during late gestation. Anim. Feed Sci. Technol.

[5] Miller Y, Collins A, Smits R, Thomson P, Holyoake P (2012). Providing supplemental milk to piglets preweaning improves the growth but not survival of gilt progeny compared with sow progeny. J. Anim. Sci.

[6] Richert B, Tokach M, Goodband R, Nelssen J, Campbell R, Kershaws S (1997). The effect of dietary lysine and valine fed during lactation on sow and litter performance. J. Anim. Sci.

[7] Nissen P, Danielsen V, Jorgensen P, Oksbjerg N (2003). Increased maternal nutrition of sows has no beneficial on muscle fibre number or postnatal growth and has no impact on the meat quality of the offspring. J. Anim. Sci.

[8] Cerisuelo A, Sala R, Gasa J, Carrión D, Coma J, Chapinal N, Baucells M (2010). Effects of extra feeding on mid-pregnancy for three successive parities on lean sows productive performance and longevity. Can. J. Anim. Sci.

[9] Vallet J.L, Miles J.R (2012). Comparison of myelination between large and small pig foetuses during late gestation. Anim. Reprod. Sci.

[10] Quiniou N, Dagorn J, Gaudre D (2002). Variation of piglets'birth weight and consequences on subsequent performance. Livest. Prod. Sci.

[11] Wolf J, Žáková E, Groeneveld E (2008). Within-litter variation of birth weight in hyperprolific Czech large white sows and its relation to litter size traits, stillborn piglets and losses until weaning. Livest. Sci.

[12] Yang Y, Heo S, Jin Z, Yun J, Choi J, Yoon S, Park M, Yang B, Chae B (2009). Effects of lysine intake during late gestation and lactation on blood metabolites, hormones, milk composition and reproductive performance in primiparous and multiparous sows. Anim. Reprod. Sci.

[13] Wu G, Bazer F, Burghardt R, Johnson G, Kim S, Li X, Satterfield M, Spencer T (2010). Impacts of amino acid nutrition on pregnancy outcome in pigs:Mechanisms and implications for swine production. J. Anim. Sci.

[14] Buitrago J, Maner J, Gallo J, Pond W (1974). Effect of dietary energy in gestation on reproductive performance of gilts. J. Anim. Sci.

[15] Sarkar D (2008). Lattice:Multivariate Data Visualization with R. Springer, New York.

[16] Wickham H (2009). Ggplot2:Elegant Graphics for Data Analysis. Springer-Verlag, New York.

[17] Bates D, Maechler M, Bolker B, Walker S (2015). Fitting linear mixed-effects models using lme4. J. Stat. Softw.

[18] Foxcroft G, Dixon W, Novak S, Putman C, Town S, Vinsky M (2006). The biological basis for prenatal programming of postnatal performance in pigs. J. Anim. Sci.

[19] Che L, Yang P, Fanf Z, Lin Y, Wu D (2013). Effect of dietary arginine supplementation on reproductive performance and immunity of sows. Czech J. Anim. Sci.

[20] Adsuar M, Fornós J, Macià R, Babot D, Álvarez-Rodríguez J (2014). Effects of pre-partum feeding level on productive performance and within-litter variation of litter body-weight from high prolific sows. Inf. Tecnic. Econ. Agrar.

[21] Muns R, Manzanilla E.G, Sol C, Manteca X, Gasa J (2013). Piglet behaviour as a measure of vitality and its influence on piglet survival and growth during lactation. J. Anim. Sci.

[22] Liu X, Wu X, Yin Y, Liu Y, Geng M, Yang H, Blachier F, Wu G (2012). Effects of dietary L-arginine or N-carbamylglutamate supplementation during late gestation of sows on the miR-15b/16, miR-221/222, VEGFA and eNOS expression in umbilical vein. Amino Acids.

